# Exogenous Melatonin as a Sleep-promoting Agent beyond its Chronobiotic Properties: A Scoping Review of its Effects on Key Sleep-wake Neurotransmitters

**DOI:** 10.2174/011570159X379708250702084616

**Published:** 2025-07-22

**Authors:** Letizia Biso, Nicola Luigi Bragazzi, Marco Bonaso, Erik Bersanetti, Sergio Garbarino, Marco Scarselli

**Affiliations:** 1 Department of Translational Research and New Technologies in Medicine and Surgery, University of Pisa, Pisa, Italy;; 2 Department of Clinical Pharmacy, Saarland University, Saarbrücken, Germany;; 3 Department of Pierpaoli Exelyas Srl, Milan, Italy;; 4 Department of Neuroscience, Rehabilitation, Ophthalmology, Genetics and Maternal/Child Sciences (DINOGMI), University of Genoa, Genoa, Italy

**Keywords:** Melatonin, sleep, sleep-wake neurotransmitters, sleep disorders, sleep-promoting agent, sleep medicine

## Abstract

**Introduction:**

Exogenous melatonin (exo-MLT) is a sleep-promoting agent that modulates key sleep-wake neurotransmitters.

**Methods:**

This scoping review analyzed 623 studies retrieved from PubMed/MEDLINE and ISI/Web of Science, applying PRISMA methodology to ensure rigorous inclusion criteria. After screening, 58 original research papers were analyzed for exo-MLT's effects on gamma-aminobutyric acid (GABA), serotonin, dopamine, glutamine, norepinephrine, epinephrine, orexin, acetylcholine, adenosine, glycine, galanin, and histamine.

**Results:**

We identified 20 studies on the GABAergic system, showing that exo-MLT enhances GABA activity through different mechanisms, promoting non-REM sleep and reducing stress-related hyperarousal. On serotonin, 16 studies revealed limited and variable effects depending on the dose and physiological conditions. Total 13 dopamine studies suggested that exo-MLT does not alter physiological dopamine turnover, restoring dopaminergic balance in pathological states. On the glutamatergic system, seven studies showed a compensatory role of exo-MLT on glutamate excitotoxicity. Six studies on norepinephrine highlighted exo-MLT's ability to regulate sympathetic activity. The orexinergic system was the focus of five studies, indicating exo-MLT's inhibitory action on orexinergic neurons, enhancing sleep quality and consolidation. Five studies investigated exo-MLT on the cholinergic system, revealing an enhancing effect on acetylcholine activity in physiological and pathophysiological conditions. Lastly, four studies exploring adenosine and glycine were inconclusive of the exo-MLT effect, while we could not find any data on histamine and galanin.

**Discussion:**

This review underscores exo-MLT's mechanisms beyond circadian regulation, offering therapeutic promise in sleep disorders associated with other neuropsychiatric conditions.

**Conclusion:**

Exo-MLT’s interactions provide insights into its safety and non-addictive characteristics, supporting its integration into personalized sleep medicine.

## 1 INTRODUCTION

**Fig. (1):**
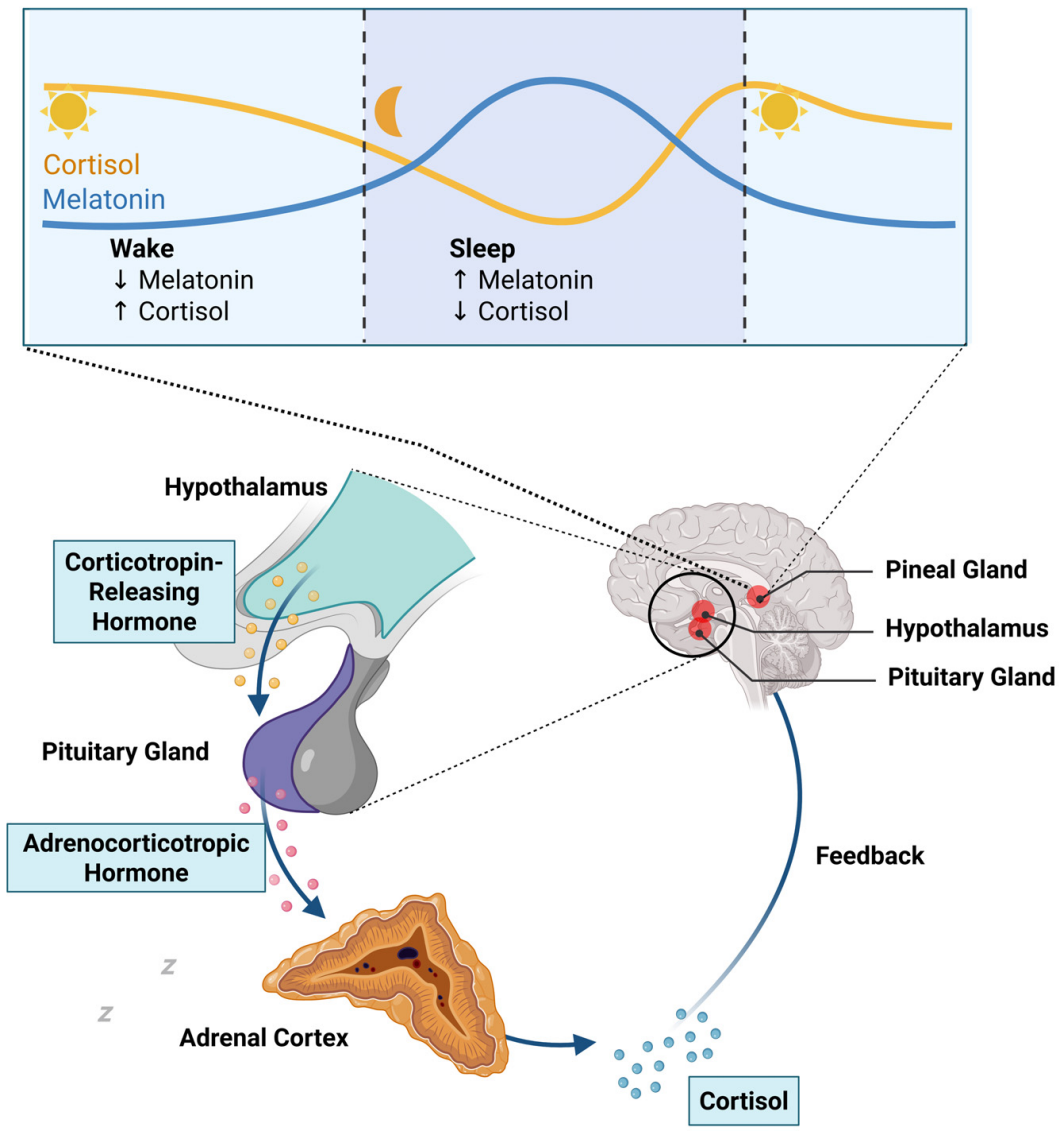
Relationship between melatonin and the hypothalamic-pituitary-adrenal axis.

Sleep is a fundamental and complex physiological process that is part of the daily sleep-wake cycle, characterized by two main phases: non-rapid eye movement (NREM) sleep and rapid eye movement (REM) sleep [1]. It follows a circadian rhythm that accumulates during wakefulness and is regulated by multiple factors, including the suprachiasmatic nucleus (SCN) and the central biological clock, the activity of which is influenced by external light cues. The SCN plays a pivotal role in regulating the synthesis of endogenous melatonin (MLT), while MLT, in turn, modulates SCN activity, forming a crucial feedback loop for maintaining circadian rhythms [2]. The light-induced activation of the SCN fosters the release of GABAergic inputs from the SCN to the paraventricular nucleus of the hypothalamus (PVN), resulting in the inhibition of the MLT synthesis in the pineal gland. The plasma level of endogenous MLT is very low and, during its nocturnal peak, it reaches the concentration in the range of 40-100 pg/ml (172-430 pM). In humans, low doses of exogenous MLT (exo-MLT) between 0.1 and 1 mg (physiological doses) produce plasma levels within the normal nocturnal concentrations (physiological doses), and they are responsible for the chronobiotic effects. On the contrary, higher doses between 1 mg to 10 mg (pharmacological doses) lead to plasma MLT concentrations from 10 to 100 times higher than the physiological peak, and they are considered optimal for the effects on sleep-related parameters [3]. Several studies have evaluated the impact of exo-MLT on endogenous MLT production, and the results are controversial. In fact, while in rats, exo-MLT increases the pineal MLT secretion through MLT receptors expressed in the SCN, in humans, endogenous MLT production was not affected by exo-MLT [4, 5]. MLT can influence hypothalamic activity, affecting the Hypothalamic-pituitary-adrenal axis, and modulating the secretion of hormones like cortisol. The administration of exo-MLT could shift MLT and cortisol secretion to earlier phases [6]. MLT exerts inhibitory control over the hypothalamic-pituitary-adrenal axis by reducing the release of corticotropin-releasing hormone (CRH) and adrenocorticotropic hormone (ACTH), thereby counterbalancing cortisol rhythms and maintaining circadian and stress-response homeostasis (Fig. **1**). As one of the most extensively studied compounds in sleep research, MLT is an indoleamine that exerts its effects through MT1 and MT2 receptors, which are coupled to inhibitory G proteins and are expressed in both the central nervous system (CNS) and peripheral tissues. These receptors modulate a variety of physiological functions, including sleep regulation [7, 8]. MT1 receptors in the CNS can be found in the hippocampus, cerebellum, locus coeruleus, substantia nigra, ventral tegmental area, nucleus accumbens, caudate, putamen, cerebral cortex, habenula, periaqueductal grey, dorsal raphe, pituitary gland, and retina [3]. Conversely, MT2 receptors are mostly expressed in the retina, olfactory bulb, forebrain, amygdala, reticular thalamus, and hippocampus [9, 10]. MT1 receptor activation primarily regulates REM sleep, while MT2 receptors are specifically linked to the promotion of NREM sleep. This functional differentiation is related to their distribution, with MT1 receptors localized in regions associated with REM sleep, such as the locus coeruleus and lateral hypothalamus, and MT2 receptors expressed in the reticular thalamus [11-13]. Beyond MLT, sleep architecture also involves other brain regions, such as the basal forebrain, the reticular activating system (RAS), the thalamus, and the hypothalamus (*e.g*., the ventrolateral preoptic area, VLPO). These areas are interconnected through an array of neurotransmitters that either promote wakefulness or sleep. Wakefulness is facilitated by glutamate (Glu), orexin/hypocretin, norepinephrine/ epinephrine, histamine, serotonin (5-HT), dopamine, and acetylcholine (ACh), while sleep is promoted by adenosine, gamma-aminobutyric acid (GABA), ACh, and MLT. Interestingly, GABAergic activity can have opposing effects depending on the neural circuits involved [14-17]. In this complex sleep-awake circuitry, the lateral hypothalamus has a prominent role and contains mainly two neuronal populations. First, the orexinergic neurons project to the cerebral cortex, basal forebrain, and the monoaminergic ascending systems [18, 19]. Secondly, neurons that contain melanin-concentrating hormone (MCH) have similar projections, but they are mostly active during sleep. If several nuclei are involved in the arousal, the most important one to turn off the arousal system is the VLPO. VLPO GABAergic neurons send inhibitory signals to monoaminergic ascending arousal systems and the orexinergic neurons of the lateral hypothalamus [20]. Intriguingly, VLPO activity is concurrently inhibited by the ascending arousal regions, thus forming a bidirectional mutually inhibitory system.-Exo-MLT has been shown to enhance GABAergic inhibitory activity, thereby promoting NREM sleep [21]. Additionally, exo-MLT may influence other neurotransmitter systems critical for wakefulness and sleep, including serotonin, noradrenaline, adrenaline, and orexin/hypocretin. This has led to the hypothesis that MLT plays a broader role in promoting sleep, extending beyond its well-established chronobiotic properties [9, 22, 23]. These neuromodulators are already targeted by various pharmacological agents used in sleep disorders, including benzodiazepines (BDZs), antihistamines, orexin/hypocretin antagonists, and certain antidepressants [24-27]. A growing body of scholarly evidence from clinical trials and meta-analysis confirmed exo-MLT efficacy in reducing sleep onset latency (SOL) and modestly increasing total sleep duration (TST), with particular benefit in sleep-wake rhythm disorders in subjects affected by insomnia and healthy volunteers [3, 28-31]. Pooled data collected in the most recent meta-analysis by Cruz-Sanabria *et al*. showed how the dose of exo-MLT of 4 mg/day and its administration 3h before bedtime obtained the best results. In patients who presented sleep disorders in comorbidity, such as Alzheimer’s and Parkinson’s disease (PD), clinical experiences could highlight a positive effect of melatonin at higher dosages [32, 33]. Compared to conventional hypnotics, especially BDZs, Z-drugs, and dual orexin receptor antagonists (DORAs), melatonin shows lower efficacy in the acute treatment of insomnia but stands out for a superior tolerability profile, less tolerance, lack of dependence, and lower impact on sleep architecture [34, 35]. Unlike BDZs and Z-drugs, chronic use of melatonin does not induce tolerance, rebound, or cognitive impairment. Furthermore, in subjects undergoing BDZ tapering, concomitant use of exo-MLT has been shown to improve sleep quality and reduce withdrawal symptoms [36, 37]. Due to its favorable safety and tolerability profile, exo-MLT has also been explored in sleep disorders associated with bipolar disorder, anxiety, autism, post-traumatic stress disorder (PTSD), epilepsy, PD, and substance abuse [38, 39]. Given these considerations, the current study aims to comprehensively evaluate the effects of exo-MLT on key neurotransmitters implicated in the sleep-wake cycle. Existing literature on this topic is fragmented and inconclusive, leaving significant gaps in our understanding of exo-MLT’s mechanisms of action. To address this, we conducted a scoping review of available evidence from preclinical (*in vitro/ex vivo* and *in vivo*) studies and clinical studies in humans.Our objectives were to analyze the mechanisms through which exo-MLT influences the activity of neurotransmitters involved in sleep regulation and to explore the therapeutic implications of these interactions, with a focus on personalized and targeted applications of exo-MLT for managing sleep disorders, both as a standalone treatment and in combination with interventions for co-occurring pathological conditions.

## 2 MATERIALS AND METHODS

This study employed a scoping review methodology, which is particularly suited to exploring broad and complex research topics where existing evidence is diverse, fragmented, or inconclusive, as in our case. Scoping reviews allow for the systematic mapping of available literature to identify key concepts, research gaps, and future directions [40]. This approach was chosen because, as previously mentioned, the effects of exo-MLT on neurotransmitter systems related to the sleep-wake cycle remain inadequately synthesized, with preclinical and clinical data often presented in a disjointed manner. The scoping review framework facilitated a comprehensive exploration of the literature across various neurotransmitter pathways, model systems, and sleep-related outcomes.

More in detail, the present scoping review leveraged the foundational methodology proposed by Arksey and O'Malley, which has since been refined by Levac *et al*., Colquhoun *et al*., and the Joanna Briggs Institute (JBI) [41-44]. These refinements enhanced methodological rigor by emphasizing iterative processes and the importance of stakeholder engagement. The steps of the Arksey and O'Malley framework, as applied to this study, were: i) identifying the research question, ii) identifying relevant studies, iii) study selection, iv) charting the data, v) collating, summarizing, and reporting results, and vi) consultation exercise.

### 2.1 Identifying the Research Question

This study presented broad yet focused questions about the role of exo-MLT in modulating neurotransmitter systems and its implications for sleep outcomes. The JBI Population-Concept-Context (PCC) framework guided the review to ensure methodological rigor and relevance. The population comprised animal models such as mice, rats, and zebrafish, as well as *in vitro*/*ex vivo* and *in vivo* systems, reflecting the preclinical focus on mechanisms of action. Human populations, if available, were included as well. The concept focused on the role of exo-MLT in modulating key neurotransmitters and their receptors in relation to the sleep-wake cycle. The context was defined as preclinical and clinical studies exploring sleep-related outcomes, including sleep architecture, latency, and quality, which are directly relevant to MLT’s therapeutic potential in sleep disorders. This specific PCC approach was chosen to align with the broad aim of synthesizing mechanistic insights while addressing the fragmented nature of existing data.

### 2.2 Identifying Relevant Studies

A comprehensive search strategy was developed to ensure the retrieval of diverse, relevant literature across preclinical and clinical settings. The search was conducted in the PubMed/MEDLINE and ISI/Web of Science databases, covering publications available from inception up to November 30, 2024. We included papers in English and Italian only. The search string was designed to capture studies investigating the effects of exo-MLT on the neurotransmitter systems implicated in sleep regulation. The string combined terms related to MLT, its target neurotransmitters and receptors, experimental model systems, clinical studies, and sleep-related outcomes using Boolean operators. Terms such as MLT, key neurotransmitters, and sleep were included, along with relevant receptor-specific and species-specific terms. This comprehensive approach ensured the inclusion of studies examining MLT’s direct and indirect effects on neurotransmitters across a range of experimental (either preclinical or clinical) models. Lastly, extensive cross-referencing was applied.

### 2.3 Study Selection

Studies were independently reviewed against predefined inclusion/exclusion criteria to ensure relevance and rigor. Titles and abstracts were independently reviewed by two evaluators (L.B. and M.B.) to determine relevance according to the predefined inclusion and exclusion criteria. In cases of disagreement, a third expert reviewer (M.S.) served as the final arbiter. Studies were included if they investigated the effects of exo-MLT on neurotransmitter systems involved in sleep regulation, utilized preclinical or clinical models, and reported sleep-related outcomes. Clinical case reports, clinical case series, technical notes and reports, non-peer-reviewed material, including conference abstracts/proceedings, theses/dissertations, books/book chapters, commentaries, editorials, letters to the editor, and opinion pieces without original data, were excluded. All types of reviews were thoroughly examined to minimize the risk of overlooking potentially relevant studies. Narrative reviews were not retained, while systematic reviews with or without meta-analyses were deemed eligible for inclusion. Full-text reviews were conducted for studies meeting the eligibility criteria by two evaluators (L.B. and M.B.), with discrepancies resolved through discussion or consultation with a third reviewer (M.S.). The entire process adhered to the PRISMA-ScR (Preferred Reporting Items for Systematic Reviews and Meta-Analyses extension for Scoping Reviews) guidelines to ensure transparency and reproducibility [45].

### 2.4 Charting the Data

Data extraction focused on key parameters, including the specific neurotransmitter(s) studied, experimental (either pre-clinical or clinical) models used, mechanistic insights into MLT’s interactions, and reported sleep-related outcomes. This approach allowed for a detailed synthesis of the available evidence, providing a clearer understanding of exo-MLT’s multifaceted role in sleep regulation.

### 2.5 Collating, Summarizing, and Reporting Results

The data were synthesized to map the evidence landscape, identify research gaps, and propose future directions, adhering to PRISMA-ScR guidelines.

### 2.6 Consultation Exercise

While not mandatory, stakeholder engagement and expert consultation were integral to refining the research question and ensuring relevance. Key contributors included E.B., the Scientific Director of a pharmaceutical company specializing in the production and sale of MLT, S.G., a neurologist and neurophysiopathologist with expertise in sleep medicine, and M.S., a neuropharmacologist with expertise in hypnotic drugs. Their insights helped align the study with practical applications and scientific rigor, further enhanced by N.L.B., a research methodologist and a biostatistician.

In conclusion, the scoping review methodology, guided by the PCC framework and informed by the PRISMA-ScR guidelines, was integral to mapping the existing landscape of preclinical and clinical research and identifying avenues for further investigation (Table **1**).

## 3 RESULTS

The initial search identified a total of 805 papers. After removing duplicates, 623 papers remained. Among these, 344 were deemed out of scope, 33 were in languages different from English or Italian, 9 lacked full-text availability, 4 were conference paper extracts, 2 were retracted, 2 were case reports, and 1 was a non-peer-reviewed journal letter. This left 227 papers for further consideration, of which 80 were reviews. Additionally, citation searching yielded 16 more relevant papers. Ultimately, 58 research papers were included in our review. The selection and inclusion process are summarized in Fig. (**2**). The main findings are outlined in the following sections and summarized in Table **2**.

### 3.1 Exo-MLT and GABA

Our research focused on *in vitro/ex vivo*, *in vivo*, and human studies to explore the relationship between exo-MLT administration and the GABAergic system. We retrieved 28 original papers through our search strategy and an additional 5 through citation searching. Of these 33 papers, 20 specifically addressed the role of exo-MLT and were included in our analysis (Table **1**, Appendix). Additionally, we identified 33 reviews discussing the relationship between MLT and GABA; however, none were systematic reviews or meta-analyses, so they were excluded.

Three studies investigated the effects of exo-MLT on the GABAergic system *in vitro*/*ex vivo*. Lowenstein *et al*. demonstrated that high concentrations of exo-MLT (100 μM) bind to the GABA-A receptor benzodiazepine site in rat cerebral cortex membranes by competing with (^3^H)flunitrazepam [46]. Another study reported that exo-MLT (1-10 μM) enhanced dorsal raphe nucleus (DRN) neuron activity by promoting Cl^-^ ion influx, thereby reducing GABAergic inhibition on DRN neurons [47].

Similarly, Cheng *et al*. found that exo-MLT (0.1-5 mM) amplified the GABAergic response in rat hippocampal neurons, an effect blocked by the benzodiazepine antagonist flumazenil but not by the MLT receptor antagonist luzindole [48].

*In vivo* studies provided further insights into the interaction between exo-MLT and the GABAergic system. Lowenstein *et al*. reported that pinealectomy in rats reduced GABA-A receptor benzodiazepine binding sites, a change reversed by exo-MLT administration (0.8-1.6 mg/kg). Furthermore, in healthy rats, five days of exo-MLT treatment increased the number of these binding sites. Similarly, minimal doses of exo-MLT restored GABA-A receptor expression in pinealectomized rats [49]. Exo-MLT was also shown to modulate GABAergic transmission in various brain regions, including the hypothalamus, cerebellum, and cerebral cortex [21, 50]. Notably, exo-MLT modulates the GABAergic system *via* MT1 receptors in the lateral hypothalamus by inhibiting hyperpolarization-activated cyclic nucleotide-gated (HCN) ion channels and *via* MT2 receptors in the thalamic reticular nucleus [51, 52].

Exo-MLT’s antioxidant and neuroprotective properties were also evident, particularly in sleep-deprived animal models. These effects were mediated through GABAergic neurotransmission, as evidenced by the blockade of exo-MLT effects by GABA-A receptor antagonists flumazenil and picrotoxin [53-56]. Exo-MLT also enhanced benzodiazepine effects, such as sleep-phase shifting, in young and old hamsters [57].

Exo-MLT has potential therapeutic applications in disease models. In an autism spectrum disorder (ASD) mouse model, chronic exo-MLT administration improved GABAergic/glutamatergic systems balance by reducing mGluR7/8 receptor and GAD67 expression, enhancing GABAergic activity [58]. In a nocturia model in female rats, exo-MLT alleviated symptoms *via* GABA-A receptor interactions [59]. For PTSD, exo-MLT reduced stress behaviors, normalized GABA and cortisol levels, and mediated effects through MT1 receptors [60]. In a rat anxiety model, exo-MLT modulated GABA/glutamate balance, reversing amygdala GABA-A-α-2 receptor subunit downregulation and NR2B-containing NMDA receptor upregulation [61].

Among human studies, one found that a high dose of exo-MLT (100 mg) combined with low-dose triazolam improved sleep microstructure and subjective sleep quality in healthy males [62]. Another study on Alzheimer’s disease patients demonstrated that exo-MLT improved sleep latency and altered EEG parameters, including alpha 1 and delta bands, and the effects were potentially mediated by the GABAergic system modulation [32].

These findings also highlighted a bidirectional interaction between GABAergic drugs and MLT secretion. GABA-A receptor agonists and allosteric modulators, such as benzodiazepines and z-drugs, affected endogenous MLT secretion differently [63-67]. For instance, chronic diazepam administration reduced MLT binding sites in the medulla-pons, an effect reversed by exo-MLT [68].

In conclusion, taken together, these results underscore the significant role of exo-MLT in modulating GABAergic system functions under physiological and pathological conditions, including anxiety, autism, and PTSD. Exo-MLT can mitigate the deleterious effects of benzodiazepines, which act as allosteric modulators of GABA-A receptors. Its mechanisms of action involve MT1 and MT2 receptors, with differential expression in GABAergic nuclei such as the lateral hypothalamus and reticular nucleus. At higher doses, exo-MLT directly interacts with GABA-A receptors, offering a multifaceted approach to modulating the GABAergic system.

### 3.2 Exo-MLT and Serotonin (5-HT)

On the interconnections between exo-MLT administration and the serotoninergic system, we were able to retrieve 39 original studies, all exclusively from the search string, along with 37 review articles. We decided not to include any of them, since they were narrative reviews. Specifically, 16 studies comprised *in vitro*/*ex vivo*, and *in vivo* experiments, while human studies were not found (Table **2** Appendix).

In an *ex vivo* research involving rat cerebral tissues, such as striatum, hippocampus, and pineal gland, there was an increase in 5-HT levels after the administration of exo-MLT [22, 69-71]. Interestingly, this effect was more pronounced in younger animals, suggesting its supplementation at earlier phases of development [70, 71]. Older research by Iyengar found that the activity of melanocytes from individuals with vitiligo was regulated by MLT and 5-HT, depending on seasonal variations in UV exposure. Higher 5-HT levels corresponded to shorter nights and longer days; conversely, higher MLT levels induced longer nights and shorter days [72]. A recent study by Yang and colleagues has demonstrated that MLT administration results in an increase of the expression of tryptophan hydroxylase 2 (TPH2), the enzyme responsible for 5-HT synthesis, and it influences wakefulness by modulating WNK-SPAK/OSR1-KNCC1 signalling in serotoninergic neurons of rodents’ DRN [47].

When analysing *in vivo* research, some differences in relation to the effects of MLT administration on the serotoninergic system could be found. However, in several animal studies on rodents or ringdoves, exo-MLT administration did not influence 5-HT levels, though it increased nocturnal rest and endogenous MLT levels [60, 73-76].

On the contrary, in a study by Haduch *et al*., the administration of 100 mg/kg of MLT increased the synthesis of 5-HT from the deacetylation of MLT-derived 5-MT mediated by CYP2D, linking the relationship between 5-HT and MLT [77]. Another study showed in male Wistar rats that the administration of MLT (1 mg/kg) counteracted the effects of both a serotoninergic antagonist (ritanserin) and a serotoninergic partial agonist [1-[2,5-dimethoxy-4-methylphenyl]-2-aminopropane or DOM), indicating the ability to modulate sleep sensitivity response indirectly by serotoninergic receptors [78].

Interestingly, in studies that compared the response to MLT in younger and older animals, there seems to be a consensus in which older animals tend to respond less to MLT treatment than younger animals [75, 76, 79].

The possible relationship between exo-MLT and the serotoninergic system was also explored in a few animal models of PTSD and Alcohol Use Disorder (AUD). In a rat AUD model, in which animals were also treated with escitalopram, the administration of MLT (40 mg/kg) reduced alcohol intake, showing a possible rationale for using MLT in the treatment of AUD, especially in the presence of sleep disturbances [80]. In another model of PTSD in mice, the administration of MLT was not able to significantly modify the 5-HT levels; however, it restored GABA and cortisol levels through the MT1 receptor, with the consequent improvement of stressful behaviours in the animals [60]. Lastly, in a murine methamphetamine-induced aggressiveness model, MLT could reverse aggressive behaviour at the dose of 5 mg/kg, and increase the concentration of 5-hydroxy-3-indolacetic acid (5-HIAA), dopamine, homovanillic acid, and reverse 5-HIAA/5-HT ratios that were lower in these aggressive animals [81].

As already mentioned, we were not able to find any study that could link exo-MLT administration and the serotoninergic system in humans.

In summary, *in vitro* data suggest that exo-MLT has some partial effects on 5-HT activity, while *in vivo* results show a limited effect on the serotoninergic system, depending on several variables, such as time of administration and dosing. Thus, we might conclude that exo-MLT does not impact the serotoninergic system as other neuromodulators, such as GABA. The mechanisms through which MLT could exert these effects could be either direct on the DRN serotoninergic neurons, which express MLT receptors, or indirect by acting on GABAergic neurons, which consequently modulate DRN nuclei.

### 3.3 Exo-MLT and Dopamine

In total, we found 37 papers and 34 reviews on the relationship between MLT and dopamine systems. None of the reviews was a systematic one or a meta-analysis, therefore, we did not include them in our review. Considering papers that examined the administration of exo-MLT and the effects on the dopaminergic system, we found 13 papers that met our requirements (Table **3**, Appendix). We found only one *in vitro* research by Schiller *et al*. in which, in a pheochromocytoma cell line (PC12), the nicotine-induced dopamine release was inhibited by MLT administration [82].

In relation to *in vivo* research, in a recent study by Chenu and colleagues, exo-MLT (40 mg/kg) did not alter the firing rate and bursting activity of ventral tegmental area (VTA) dopamine neurons and other monoaminergic neurotransmitters by itself, but only in combination with 5HT2b and 5HT2c antagonists, which explains the superiority of agomelatine compared to MLT regarding antidepressant properties [73]. The other two studies on rodents have also shown that although there is a positive effect of MLT on sleep duration, MLT did not alter dopamine or other catecholamine levels in the brain [60, 83]. In addition, in one study on primates in pregnant rhesus macaques exposed to constant light, Matsumoto and Tucsay found that MLT-infused administration (0.2 pg/kg/h) during the night hours did not modify dopamine (nor epinephrine) levels [84]. Interestingly, in mice, the co-administration of MLT and quinpirole, a D2 and D3 receptor agonist, exerted a potentiation on MLT effects on sleep, suggesting that D2 receptor activation could improve sleep quality in the presence of exo-MLT [50].

In an addiction model in rats, animals treated repeatedly with cocaine showed an increase of anxiety and cAMP in the nucleus accumbens, which were both reverted by MLT pre-treatment, showing an anxiolytic-like effect of MLT [85]. In addition, MLT by itself did not change animal behaviour in the free-choice self-administration setting, underlying its safety and low dependence liability. Similar data were reported for mice treated with methamphetamine (MA), which is considered a model of addiction and neurotoxicity as well. MLT pre-treatment (5 mg/kg) reduced MA-induced aggressive behaviours by normalizing the acute changes in the serotoninergic and dopaminergic system and probably counteracting cellular toxicity [81].

Considering other pathologies, in a PD model of zebrafish treated with N-methyl-4-phenyl-1,2,3,6-tetrahydropyridine (MPTP), causing disruption of the dopaminergic system and day/night cycles, the administration of MLT was able to fully restore the normal circadian system [86]. Similarly, in a rotenone-induced model of PD in rats, Bassani *et al*. found that MLT could restore dopamine levels, most likely by inhibiting monoamine oxidase (MAO), which could also account for some antidepressant-like activity of MLT observed in this study [69]. Recently, two different groups in rodents treated with D2 receptor antagonists investigated whether the administration of MLT could improve extrapyramidal symptoms. In one study, MLT was able to reduce hypokinesia induced by haloperidol only in adult rats, where MLT levels are supposed to be reduced, while in the second study, MLT at 10 mg/kg reduced hypokinesia in wild-type mice and MT1 receptor knockout mice, demonstrating the relevance of MT2 receptors [87, 88].

Taken together, these studies demonstrated that MLT did not alter the normal physiological turnover of the dopaminergic system, underlining its low risk for causing addictive behaviours. This is relevant if we compare it to other hypnotic drugs, such as benzodiazepines [89]. Conversely, in animal models representing pathological conditions where the dopaminergic system is defective, such as drug abuse, PD, and tardive dyskinesia, exo-MLT is able to reverse the neurochemical and behaviour changes. For instance, in animal models of drug addiction, MLT demonstrated anxiolytic properties and rescued the disruption of the day-night cycle. In relation to the mechanism, it is possible that exo-MLT could exert a direct effect on the dopaminergic circuitry mostly through MT1 receptors, but also indirectly through the GABAergic interneurons.

### 3.4 Exo-MLT and Glutamate

Glutamate (Glu), the principal excitatory neurotransmitter, plays a critical role in sleep/wake regulation. The median preoptic glutamatergic neurons project to brain regions that are important in the wake-sleep cycle, like the VLPO, lateral hypothalamic area, ventrolateral periaqueductal gray matter, tuberomammillary nucleus, locus coeruleus, and parabrachial nucleus, having opposite roles in sleep regulation [90]. For the purpose of this review, we retrieved 7 original research papers (Table **4**, Appendix) and 18 reviews on the relationship between exo-MLT and Glu. We excluded the reviews because none of them was a systematic one. The only *in vitro* study from Evely *et al*. (2016) demonstrated that exo-MLT increased glutamatergic synaptic firings in neurons derived from the lateral habenula of rats [91]. Concerning the other six studies on animal research, one by Tardito and colleagues demonstrated that exo-MLT (40 mg/kg) was not able to reduce the stress-induced Glu increase in the rats' prefrontal and frontal cortex [92]. On the contrary, in an anxiety model induced by sleep deprivation, exo-MLT administered by intraperitoneal perfusion (15 mg/kg) was able to counteract NR2B-containing N-methyl-D-aspartate and GluR1 receptors up-regulation, indicating its ability to regulate excitatory neurotransmission in an animal stress model [61]. In a mouse model, Mao and colleagues administered intraperitoneally exo-MLT (0.5 mg/day), showing that MLT was able to reduce necroptosis-related Glu metabolism pathway [93]. An improvement by exo-MLT administration was also obtained in an ASD mouse model, as a consequence of a change in expression in mGluR7/8 receptors and glutamate decarboxylase enzyme GAD67 in the PFCs [58]. Lastly, in a recent study by Zhao *et al*., researchers demonstrated that exo-MLT administration combined with exercise led to an increased expression of ionotropic GluR2 and a decreased Glu excitotoxicity, with the ability to ameliorate sleep disorders and synaptic plasticity [94]. These results underline the capability of exo-MLT to counteract Glu excitotoxicity in models where the glutamatergic system is hyperactive, such as ASD and neurodegenerative disorders.

### 3.5 Exo-MLT and Norepinephrine/Epinephrine

Overall, our search retrieved 30 original studies from the search string and 2 studies from the citation search. Of these, only 6 specifically investigated the effect of exo-MLT on norepinephrine and epinephrine and were included in our review (Table **5**, Appendix). We also identified 38 reviews that mentioned a potential relationship between exo-MLT and norepinephrine/epinephrine. However, as these studies were not systematic reviews or meta-analyses, they were not considered eligible for inclusion.

Chenu *et al*. explored the effect of chronic MLT administration (40 mg/kg/day) for 14 days in Sprague-Dawley rats, finding that there were no significant changes in the frequency or pattern of firing of noradrenergic neurons in the locus coeruleus [73]. Similarly, on gestating rhesus macaques under constant light exposure, exo-MLT (0.2 µg/kg/h) failed to restore the epinephrine rhythm or norepinephrine secretion [84].

On the contrary, other research suggests a more complex and nuanced role in the regulation of the noradrenergic axis. Of particular interest is the study by Chuluyan *et al*., in which a single injection at the beginning of the dark phase of exo-MLT (300 µg/kg) reduced the turnover of norepinephrine by 40% after 3 hours. Additionally, *in vitro* experiments showed that MLT (10^-8^-10^-6^ M) inhibits depolarization-induced norepinephrine release only during the night, while at very low concentrations (0.5 pM) it reduced the uptake of norepinephrine with a maximum effect at the end of the light period [95]. Another evidence comes from the study by Pazo *et al*., who showed that MLT administration (30 μg/day) for 11 days increased the amplitude of the 24-hour circadian rhythm of pineal norepinephrine content by 120% in young rats and by 52% in old rats [71]. In line with these results, Brusco *et al*. demonstrated that the treatment of rats with MLT (10 or 100 μg) for 17 days enhanced norepinephrine synthesis, especially in old animals [96]. Lastly, Xu and colleagues explored the effect of MLT in a mouse model of PTSD. The administration of a medium dose (2 mM, approximately 2.32 mg/kg) reduced epinephrine and norepinephrine levels and alleviated PTSD-like symptoms. This effect was blocked by the MT1/2 receptor antagonist (luzindole), indicating the involvement of these receptors in MLT’s action [60].

These results together have shown that exo-MLT can have very different effects on the noradrenergic systems, either inhibitory or stimulatory, depending on the animal’s age, animal model, circadian rhythms integrity, time of administration, and dose. In one paper, exo-MLT was able to reduce norepinephrine, and in an animal model of PTSD, it decreased norepinephrine levels by alleviating PTSD-like symptoms and improving sleep quality. These preliminary data show a potential effect of exo-MLT on promoting relaxation and sleep, when the sympathetic system could be hyperactive; however, more studies are strongly required to confirm this mechanism.

### 3.6 Exo-MLT and Orexin/Hypocretin

The interaction between exo-MLT and the orexinergic system is a field of growing interest for understanding the mechanisms underlying the regulation of sleep and circadian rhythm. In the literature, we retrieved a total of 11 original articles, 9 from the search string and 2 from citation searching, and 24 review articles. Although many reviews mentioned the relationship between the melatonergic and orexinergic systems, none of them were systematic reviews or meta-analyses and therefore were not included in our study. Of these original studies, only 5 *in vivo* specifically explored the role of exo-MLT and were included in our review (Table **6**, Appendix). Surprisingly, we did not find *in vitro* studies or in humans.

Among these, one of the most significant contributions was provided by the study conducted by Appelbaum *et al*. in the zebrafish model. The authors identified a specific interaction between the melatonergic and orexinergic systems illustrated by the hypersensitivity to MLT in orexin/hypocretin receptor-deficient mutant fish (hcrtr^−/−^), which represents a model of narcolepsy. In particular, the administration of low doses (1 μM) of exo-MLT showed an increase of sleep duration and sleep consolidation by 30-40% in orexin/hypocretin receptor-deficient mutant fish (hcrtr^−/−^) compared to the wild-type siblings. This effect was specific to MLT, since four other classes of hypnotics (barbiturates, benzodiazepines, antihistaminergic, and α2-adrenergic agonists) did not show differences between hcrtr^−/−^ fish and their wild-type siblings. These data suggest the existence of a functional interaction between melatonergic and orexinergic systems for regulating sleep and circadian rhythms [97].

In a subsequent study, the same group in the zebrafish model observed that increased orexinergic activity led to reduced sensitivity to exo-MLT and attenuated its sleep-promoting effects. Thus, the authors confirmed the crosstalk between orexinergic neurons and MLT, with opposite sensitivity to MLT administration depending on orexinergic neuron activity [98].

A few years later, Sharma and colleagues showed that exo-MLT can significantly reduce the activity of orexinergic neurons in the perifornical nucleus of the lateral hypothalamus of rats, with a 66% reduction in neuronal activation mediated by MLT's interaction with MT1 receptors expressed on orexin/hypocretin neurons. This effect was accompanied by a 43.7% increase in NREM sleep and a 12.3% reduction in wakefulness. Furthermore, infusion of MLT receptor antagonists, such as luzindole, demonstrated an increase in wakefulness and a reduction in NREM and REM sleep, supporting the role of endogenous MLT as well [23].

Another research group provided further evidence for the mechanisms of action of exo-MLT on the orexinergic system. Infusion of exo-MLT in zebrafish exposed to 17β-trenbolone, a substance that induces hyperactivation of the orexinergic system, significantly reduced the expression of the orexin/hypocretin receptor type 2 (*HCRTR-2*) in the lateral hypothalamic nucleus and thus restored a normal sleep-wake cycle, suggesting a direct regulatory effect of MLT on orexinergic transmission [25]. More recently, Cakir *et al*. (2023) observed that exo-MLT did not significantly alter orexin-A levels in REM sleep-deprived rats. However, the authors noted an impact on other endocrine systems, such as leptin and nesfatin-1, which are involved in the regulation of sleep and metabolism, suggesting that MLT may influence sleep and energy balance through multiple physiological pathways [99].

Overall, the few studies available suggest that exo-MLT exerts an inhibitory action on the orexinergic system either in physiological conditions or in pathophysiological settings, in which the orexinergic activity is altered. However, the number of studies is still limited, and investigations are conducted especially in zebrafish and rodents, thus requiring additional research in order to confirm these findings. The mechanism of action of exo-MLT is mostly mediated by MT1 receptors expressed in the orexinergic neurons of the perifornical nucleus of the lateral hypothalamus, but is also indirect through the GABAergic system, which consequently alters the orexinergic activity.

### 3.7 Exo-MLT and Acetylcholine (ACh)

Acetylcholine (ACh) is a neurotransmitter involved in various processes of the CNS, including the regulation of REM sleep [100]. With our search string, we found 14 narrative reviews (which were therefore excluded from our review) and two research papers. In addition, we could retrieve 3 more papers from citation searching (Table **7**, Appendix). In one study by Cardinali *et al*. (1998), exo-MLT at 10 and 100 μg dosages was able to increase daily oscillations of 3[H]acetylcholine production from 3[H]choline in the submaxillary lymph nodes of both young and old rats [101]. Similarly, in another study at the same dosages, exo-MLT increased the amplitude of nocturnal ACh synthesis and norepinephrine in old rats [96]. Within this line of research, two studies found that exo-MLT (3 and 100 μM) was able to increase ACh concentrations in the nucleus accumbens [92], but it disrupted at 200-500 μm extracellular Ach in the rat medial prefrontal cortex (MPC) [102]. Both researchers linked ACh transmission to SCN control, mediated by exo-MLT. Lastly, one recent paper by Tancheva found that MLT (20 mg/kg) increased ACh levels by 54% in 6-OHDA-treated rats, demonstrating promising antiparkinsonian properties [103]. Although the number of retrieved articles is low, these results indicate that exo-MLT administration is able to increase ACh levels in physiological and pathophysiological conditions.

### 3.8 Exo-MLT and Adenosine

Regarding the relationship between exo-MLT and adenosine, our search string yielded 2 original papers and 10 reviews, while 3 additional articles were identified through citation searches. In total, only 3 papers based on *in vitro*/*ex vivo* research met the criteria for inclusion in our analysis (Table **8**, Appendix).

In the paper by von Gall and colleagues on the hypophyseal pars tuberalis (PT) hamsters, MLT (1 µg/g) administrated pre-pinealectomy preserved the expression of *Per1* mRNA and PER1 protein in the PT. The effect of exo-MLT was mediated by adenosine A2b receptor signalling and clock-gene expression [104]. In another work, in the forebrain tissue of juvenile male mongrel albino rats, the effects of 1 mg/kg of exo-MLT were not able to change 5’-nucleotidase activity, while in an acute hypoxic setting MLT increased its levels by 18%. Moreover, in forebrain tissues from normoxic animals kept in constant darkness, exo-MLT promoted adenosine synthesis by 23% [105]. Lastly, Cao and colleagues found that in neural retinas adapted to darkness, the perfusion of 100 nM MLT during the subjective day did not have any effects on the content of adenosine [106]. These contradictory results do not let us draw definitive conclusions on the actual relationship between exo-MLT and the adenosinergic system.

### 3.9 Exo-MLT and Glycine

Glycine is a non-essential amino acid neurotransmitter with neuromodulatory properties in the CNS, and its supplementation has been linked to potentially improved sleep quality [107]. To examine the possible correlations between exo-MLT and glycine, we included this amino acid in our search string, but we could only retrieve three narrative reviews (that did not meet our inclusion criteria) and only one research paper. In transgenic mice with deficient glycine receptor function, a model of REM sleep behavior disorder (RBD), exo-MLT could improve RBD, in terms of motor activation, behavior, and sleep. However, these findings indicate that the relationship between glycine and exo-MLT is still unknown, and further studies are necessary.

### 3.10 Exo-MLT and Galanin

Preoptic galanin neurons have been implicated in the regulation of sleep homeostasis [108]. We found no papers on the relationship between exo-MLT and galanin from our search string. However, we were able to find an article from grey literature, which, after full-text assessment, was included in our scoping review. In this study, 3 mg/10 kg body weight of exo-MLT, administered to intact and castrated dogs daily for one month, resulted in a decrease in galanin levels, which was more pronounced in the castrated animals [109]. However, the presence of only one paper clearly shows the lack of research in relation to the exo-MLT’s effect on galanin production [109].

### 3.11 Exo-MLT and Histamine

Despite the well-documented roles of histamine in regulating wakefulness, surprisingly, our review found no studies that met the inclusion criteria for analysing the interaction between exo-MLT and histamine. Histamine plays a critical role in maintaining vigilance through H1 and H3 receptor activation [110], and antihistaminergic medications like hydroxyzine and mirtazapine are used to treat sleep disorders [111, 112]. A review of the available research yielded only six original papers and nine narrative reviews mentioning both MLT and histamine, including two studies identified through citation searches. However, none of these studies met the criteria for inclusion in our analysis, underlining the strong need for further research on this topic.

## 4 DISCUSSION

**Fig. (3):**
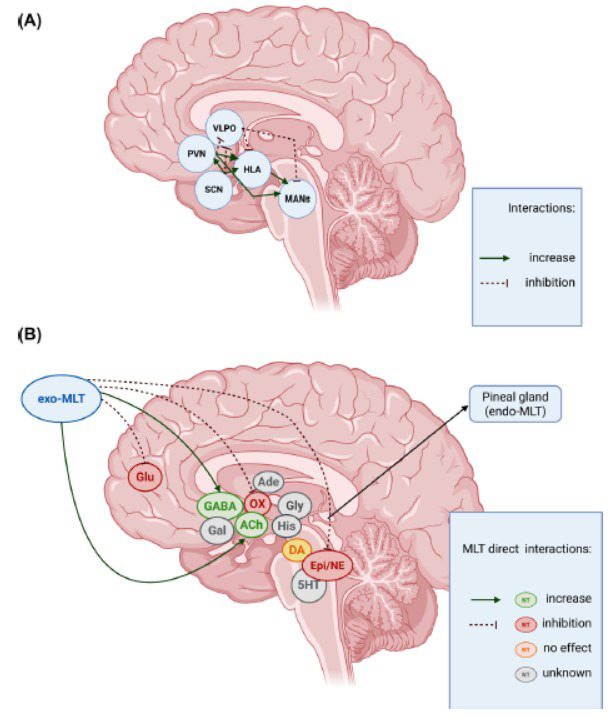
Neuroanatomical and neurochemical interactions regulating sleep-wake cycles and the effects of melatonin (MLT). (**A**) Schematic diagram of core brain nuclei involved in the regulation of sleep-wake states, including the ventrolateral preoptic nucleus (VLPO), suprachiasmatic nucleus (SCN), hypothalamic lateral area (HLA), monoaminergic nuclei (MANs), and the paraventricular nucleus (PVN). Green arrows indicate excitatory/increasing interactions, while red dashed lines represent inhibitory influences. The SCN, as the master circadian pacemaker, promotes arousal through HLA and PVN activation, while VLPO exerts inhibitory control over wake-promoting regions to facilitate sleep onset. (**B**) Direct effects of exogenous MLT (exo-MLT) on key neurotransmitters (NTs) involved in sleep regulation. Green solid lines denote MLT-induced increases, red dashed lines indicate inhibition, orange borders reflect no effect, and grey outlines denote unknown effects. The following NTs are represented: GABA (gamma-aminobutyric acid), Glu (glutamate), Gal (galanin), ACh (acetylcholine), OX (orexin/hypocretin), Ade (adenosine), Gly (glycine), His (histamine), DA (dopamine), Epi/NE (epinephrine/norepinephrine), and 5HT (serotonin).

Besides the well-known effect as a chronobiotic agent, exo-MLT has been proposed to be endowed with hypnotic properties, improving several aspects of the sleep architecture, such as sleep onset latency, number of awakenings, sleep quality, and total sleep time. The circadian control of sleep spindles relies on hormonal and neuropeptidergic signaling pathways. In humans, exo-MLT oral administration has been shown to increase sigma power density [113]. Similarly, in mice studies, systemic infusion of MLT agonists stimulated burst firing in the thalamic reticular nucleus *via* activation of MT2 receptors, enhancing sigma band activity. These findings suggest that MLT directly influences the cellular processes responsible for spindle generation [52, 114]. In the sleep architecture, several nuclei such as basal forebrain, the RAS, thalamus, and hypothalamus are determinant in promoting wakefulness or sleep, either in the REM or NREM phases. The interactions between these areas are mediated through various neuromodulators (Table **2**). Among these, orexin/hypocretin, Glu, noradrenaline, adrenaline, histamine, 5-HT, dopamine, and ACh promote wakefulness, while adenosine and glycine induce sleep, and GABA can have opposite effects depending on the circuitry involved [14, 15, 115]. With this data, we decided to investigate the effect of exo-MLT on the most relevant neurotransmitters mentioned above, through the rigorous analysis of the scoping review, allowing for a systematic mapping of available literature to identify key concepts, research gaps, and future directions. On this topic, surprisingly, the existing literature is fragmented, inconclusive, and lacks systematic studies, leaving significant gaps between the putative hypnotic properties of exo-MLT and its basic mechanism of action on the main neurotransmitters that regulate sleep architecture. This scoping review was mostly based on preclinical data, *in vitro/ex vivo*, and *in vivo*, whereas information about humans was somewhat limited due to the paucity of available data. The studies analyzed in this review were highly heterogeneous in terms of animal models to measure sleep-related parameters, dosages, and routes of administration of exo-MLT. For instance, the doses used of exo-MLT were very high and very different, in the pharmacological range, varying from 100 nM to 5 mM *in vitro* [48, 106], from 0.25 to 150 mg/kg *in vivo* [55], and at 5 mg and 100 mg dosage in humans [32, 62]. A summary of the basic relationship between exo-MLT and the neurotransmitters that we have analyzed can be found in Fig. (**3**). From our analysis, it emerged that the most important neurochemical system responsible for exo-MLT action is the GABAergic system, especially at the level of the lateral hypothalamus and the ascending RAS, which express MLT receptors. The GABAergic nuclei of the VLPO regulate mostly the NREM sleep, but other nuclei influence the REM phase as well (Fig. **3A**) [14]. Importantly, GABA is relevant for controlling the activity of several other neurotransmitters such as orexin/hypocretin, histamine, noradrenaline, adrenaline, and serotonin, underlining its central role in the wake-sleep cycle. This means that the effect of exo-MLT on GABA goes beyond the GABAergic system, and it influences other neurotransmitters that are important for wakefulness and sleep (Fig. **3B**). In addition, considering the relevance of GABA in several pathologies such as anxiety, autism, PTSD, and epilepsy, the use of exo-MLT for sleep disorders related to these conditions evidently seems a valuable option. On the contrary, the chronic use of benzodiazepines can alter sleep microstructure (Cyclic Alternating Pattern, CAP), with a suppression of slow-wave power activity in NREM sleep, especially in individuals with a history of benzodiazepine misuse [116]. Interestingly, some studies have shown that some negative effects induced by benzodiazepine administration in animal models, such as the reduction of MLT receptors, were reversed by exo-MLT [68], suggesting the potential capability of exo-MLT to rescue some benzodiazepine adverse effects. One possibility to explain the differences between MLT and benzodiazepines comes from their mechanisms of action. While benzodiazepines are powerful positive allosteric compounds acting on the GABA-A receptor, exo-MLT seems to have a more subtle and distinct action influencing GABA turnover and activity, though different mechanisms [56, 117]. Besides the GABAergic system, exo-MLT is capable of influencing the orexinergic system activity, which has recently received much attention in the light of the discovery of a new hypnotic drug such as daridorexant, an antagonist of orexin receptors [118]. On this subject, exo-MLT mostly exerts an inhibitory action on the orexinergic activity directly through MT1 receptors and indirectly through the GABAergic system. Importantly, in an animal model of narcolepsy where the orexinergic system is altered, exo-MLT was able to partially recover this dysfunction by improving sleep duration and sleep consolidation, with potentially relevant consequences for the treatment of this disorder [97]. As a matter of fact, the effect of exo-MLT on the orexinergic system highlights the relevance of this neurotransmitter in the MLT regulation of sleep and circadian rhythm. In addition to sleep regulation, melatonin may also contribute to the control of metabolic disorders frequently associated with sleep-wake rhythm disorders. Since sleep deprivation is a known risk factor for obesity and metabolic dysfunction, sleep improvement and action on the orexinergic system represent possible mechanisms through which melatonin may exert beneficial effects on metabolism [99, 119]. Indeed, melatonin is able to modulate orexinergic and anorexinergic neuropeptides, and its inhibitory action on orexin, mediated at least in part by MT1 receptors, could contribute not only to sleep regulation but also to energy balance [99, 119, 120]. This suggests that, in humans, exo-MLT could improve metabolism in patients with sleep disorders associated with metabolic dysfunction, such as obesity or diabetes. On the other neurotransmitters that are important for wakefulness, such as histamine and noradrenaline, the data are inconclusive. Surprisingly, we did not find any paper on histamine, whereas at least two papers analyzed the activity of exo-MLT on the noradrenergic system. The relevance of excessive arousal due to hyperactive noradrenergic tone is well established as a relevant factor in sleep disorders [121, 122]. In one paper, exo-MLT was able to reduce noradrenaline release, suggesting a possible inhibitory effect that could have positive repercussions on sleep quality [95]. In the second one, in an animal model of PTSD, exo-MLT reduced norepinephrine levels by alleviating PTSD-like symptoms and improving sleep quality [60]. All the preliminary data underline that exo-MLT could promote relaxation and sleep, especially in stressful situations, where the sympathetic system could be hyperactive. However, exo-MLT effect on the noradrenergic systems is influenced by several factors, including the animal model utilized, animal age, dose, and experimental context. In fact, even if a few studies demonstrated exo-MLT inhibitory activity, in one study with aged animals, exo-MLT stimulated noradrenaline synthesis, regulating circadian rhythm. This might suggest that the exo-MLT effect on the noradrenergic systems is influenced by age in humans, as well. Regarding the interactions between exo-MLT and histamine, studies are surprisingly missing; however, experimental data suggest that endogenous MLT influences histaminergic activity *via* NF-κB signaling and mast cell activation, both of which are involved in circadian regulation and inflammatory responses [123]. In this context, MLT has been shown to inhibit mast cell degranulation and reduce peripheral inflammation, potentially contributing to improved sleep quality in inflammatory conditions such as atopic dermatitis, where histamine-induced nocturnal symptoms often disrupt sleep [124]. These observations raise the hypothesis that exo-MLT may exert similar modulatory effects on central histaminergic neurons involved in arousal. In relation to two other important neurotransmitters for cognition that are implicated also in neurodegenerative disorders, such as acetylcholine and glutamate, a few data were available. Five studies investigating the cholinergic system revealed that exo-MLT is able to increase acetylcholine activity in physiological and pathophysiological conditions. Similarly, exo-MLT also has a compensatory role in neurodegenerative models induced by glutamate excitotoxicity, underlying its potential neuroprotective characteristics. Unexpectedly, we found only 3 papers on the interactions between exo-MLT and adenosine, whose characteristics as a physiological sleep inducer are well established. The results were mixed and inconclusive; thus, further investigation is warranted. There was lack of evidence for glycine, with only one study, and for galanine, no study was found. Regarding the effect on dopaminergic activity, exo-MLT does not alter dopamine turnover and release, on the contrary, benzodiazepines generally increase the dopaminergic tone [125]. This is relevant in terms of the risk of addiction, supporting the use of exo-MLT as an alternative to benzodiazepines. In addition, in rats treated with cocaine, which caused an increase in anxiety, exo-MLT reduced anxiolytic-like behaviours [85]. On the contrary, in an animal model of PD with consequent alterations of the day/night cycles, exo-MLT restored the circadian rhythm and dopamine levels, probably through its neuroprotective effect [84]. Lastly, investigating the effect of MLT on the serotoninergic system, we found a limited and controversial effect of exo-MLT, underlining how this neurotransmitter is probably less relevant compared to the others for explaining MLT activity. In summary, the hypnotic effects of exo-MLT are mediated through its inhibition of the orexinergic neurons of the lateral hypothalamus, through direct and indirect mechanisms (VLPO GABAergic activation). In addition, exo-MLT activation of the VLPO GABAergic neurons leads to the inhibition of the monoaminergic ascending arousal systems, especially of the noradrenergic locus coeruleus, the serotoninergic raphe, and the dopaminergic VTA nuclei. Surprisingly, the effect of exo-MLT on two important neurotransmitters for the sleep/wake cycle, such as histamine and adenosine, has not been sufficiently investigated, so further studies are strongly required.

### 4.1 Safety Profile and Pharmacokinetics of exo-MLT

Our scoping review mostly found preclinical studies, with very limited research on humans on this topic. However, exo-MLT tolerability and safety are well documented in several clinical studies in the short and long term. Two systematic reviews and meta-analyses confirmed its general safety in oral formulations across a wide dosage range (0.3 mg to 1600 mg), with mild adverse effects such as dizziness, headache, and hypothermia, which typically resolved within days [126, 127]. In very rare cases, more serious effects were reported, such as fatigue, mood swings, agitation, disturbed sleep, palpitations, or irritated skin. However, these effects disappeared in the first days of treatment, and they were usually exacerbations of pre-existing conditions. In relation to exo-MLT interactions with other drugs, some reports have noted potential interactivity with cardiovascular drugs (*e.g*., nifedipine), antidepressant medications (*e.g*., antidepressants inhibiting CYP1A2), and z-compounds (*e.g*., zolpidem). Possible effects on reproductive function and glucose tolerance in the presence of specific genetic mutations were also reported [127-130]. When considering patients over 65 years of age, exo-MLT seems to be a safer alternative to other commonly prescribed medications, like BDZs or antipsychotics. In a recent review, Tuft *et al*. reported a generally favorable profile of oral exo-MLT, even in older patients with neurodegenerative conditions [131]. In children and adolescents, the use of exo-MLT has been mainly studied in the context of ASD and Attention-Deficit/Hyperactivity Disorder (ADHD). In general, exo-MLT administration in children and adolescents with ASD or ADHD is safe, with a small risk of increased enuresis, drowsiness, dizziness, headache, and gastrointestinal disturbances. These results indicate a good safety profile, which has been confirmed in long-term studies [132]. Lastly, it is important to mention that a recent systematic review and meta-analysis have analyzed the safety of the use of exo-MLT at dosages above 10 mg/day, in different conditions beyond the treatment of sleep disorders. Most of the studies involved oral use of exo-MLT, with only a few studies that considered sublingual or other routes of administration [133]. Prolonged-release formulations have been shown to be more effective in improving sleep continuity and reducing night-time awakenings. Furthermore, the route of administration significantly influences pharmacokinetics: sublingual administration shows greater bioavailability compared to conventional oral administration, due to the avoidance of first-pass metabolism. The transdermal route, although presenting a slower and more variable absorption, can be useful to ensure prolonged release [134-136].

### 4.2 Strengths and Limitations

Strengths of this scoping review include its methodological rigor, characterized by a systematic and comprehensive search of the literature and strict adherence to established protocols and guidelines such as the PRISMA-ScR framework. Furthermore, this review represents the first comprehensive effort to explore the interactions between exo-MLT and key neuromodulators, providing a foundational synthesis of evidence in this critical area of sleep research.

Limitations of this review stem from the predominance of studies conducted in animal models, with a scarcity of data from human research. The studies analyzed in this review were highly heterogeneous in terms of animal models to measure sleep-related parameters, dosages, and routes of administration of exo-MLT. While these preclinical studies provide valuable insights and possess strong translational potential, their findings must be validated and further investigated in robust human studies to ensure their applicability and relevance to clinical practice.

## CONCLUSION AND FURTHER PERSPECTIVES

In conclusion, this scoping review has demonstrated the multifaceted properties of exo-MLT that go beyond its regulation on the circadian sleep-wake cycle, supporting its integration into personalized sleep medicine. Exo-MLT is able to deeply influence several neurotransmitters that are key regulators of the sleep architecture. This might explain the capability of MLT to improve several aspects of sleep, especially when the balance of certain neuromodulators is altered. As a consequence, in sleep disorders associated with PD, epilepsy, anxiety, narcolepsy, drug abuse, autism, and PTSD, the use of exo-MLT looks promising, and it should encourage clinical trials in order to establish its use in clinical practice, considering also different doses and times of administration. Considering the very high heterogeneity of the included studies, a more restricted analysis could be helpful in order to perform subgroup analyses on specific parameters that might influence exo-MLT effects on the different neurotransmitters (*e.g*., dosage).

## AUTHORS’ CONTRIBUTIONS

The authors confirm their contribution to the paper as follows: analysis and interpretation of results: NLB; draft manuscript: LB, MB, EB, SG, Conceptualization: MS. All authors reviewed the results and approved the final version of the manuscript.

## Figures and Tables

**Fig. (2):**
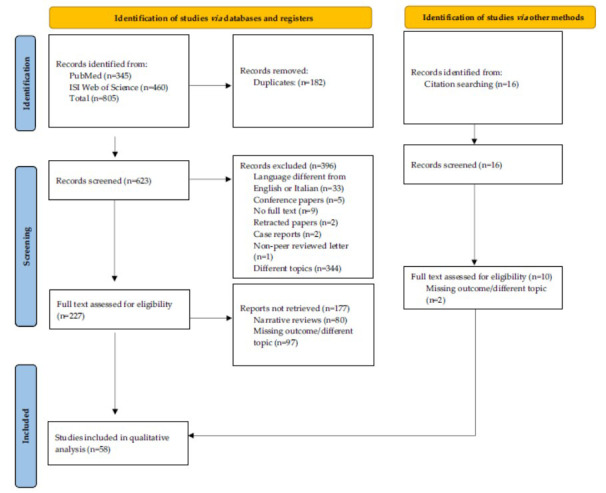
Pictorial flowchart of the selection and inclusion process adopted in the present scoping review.

**Table 1:** An overview of the methodology applied in the present study.

**Search Item**	**Details**
Databases	PubMed/MEDLINE and ISI/Web of Science
Population-Concept-Context	• *Population*: animal models such as mice, rats, and zebrafish, as well as *in vitro/ex vivo* and *in vivo* systems and human populations • *Concept*: the role of exo-MLT in modulating key neurotransmitters and their receptors in relation to the sleep-wake cycle • *Context*: preclinical and clinical studies exploring sleep-related outcomes, including sleep architecture, latency, and quality
Search string	Melatonin* AND (benzodiazepine* OR “benzodiazepine receptor*” OR acetylcholin* OR noradrenalin* OR adrenalin* OR histamin* OR GABA* OR “gamma-aminobutyric acid” OR dopamin* OR orexin* OR hypocretin* OR adenosin* OR glycin* OR galanin* OR glutamat* OR “acetylcholinergic receptor*” OR “cholinergic receptor” OR “noradrenergic receptor*” OR “adrenergic receptor*” OR “histaminergic receptor*” OR “GABAergic receptor*” OR “gamma-aminobutyric acid receptor*” OR “dopaminergic receptor*” OR “orexinergic receptor*” OR serotonin OR “serotoninergic receptor*” OR “adenosinergic receptor*” OR “glutamatergic receptor*”) AND (animal* OR mice OR murine OR “animal model*” OR zebrafish OR rat OR rats) AND (sleep* OR insomnia)
Time filter	From inception until 30 November 2024
Language filter	English and Italian
Further items	Cross-referencing

**Table 2:** Direct and indirect effects of exo-MLT on neurotransmission implicated in sleep-wake regulation.

Neurotransmitter	Brain Region(s)	Function in Sleep-Wake Regulation	Direct Effects of exo-MLT	Indirect Effects of exo-MLT
GABA	VLPO, hypothalamus, thalamus	Induces and maintains NREM sleep; inhibits arousal-promoting systems	↑ GABA tone *via *MT1 (VLPO, hypothalamus)	GABA-A activation → inhibition orexin, NA, 5-HT, histamine
Orexin	Perifornical lateral hypothalamus (LH/Pef)	Stabilizes wakefulness and maintains arousal	Direct inhibition of orexinergic neurons *via *MT1	Further suppression *via *MT1-mediated GABA release
Serotonin	Dorsal raphe nucleus (DRN), SCN	Modulates circadian rhythms and sleep transitions	Inconclusive: possible MT1/MT2 effect on DRN, but variable	Possibly indirect inhibition *via *GABAergic activation (MT1 → GABA → 5-HT)
Dopamine	Midbrain (VTA), Nucleus accumbens,	Promotes wakefulness, motivation, and REM initiation	No effect on DA turnover in physiological conditions	Indirect inhibition *via * GABAergic activation (MT1 → GABA → DA)
Norepinephrine/ Epinephrine	Locus coeruleus, hippocampus, amygdala	Supports alertness and stress response; suppressed during sleep	Inhibition of NE/EPI release *via *MT1/MT2 in selected models (dose- and context-dependent)	Possibly *via *GABAergic modulation; not consistently demonstrated
Acetylcholine	Basal forebrain Nucleus accumbens, prefrontal cortex	Active during REM sleep and cognitive arousal	↑ ACh observed, but mechanism unknown	Not reported
Glutamate	Cortex, hippocampus, cerebellum	Promotes arousal and REM transitions	↓ Glutamate (MT1/MT2 involvement unknown; possible antioxidant mechanisms)	↓ Glutamate levels *via* GABAergic modulation
Histamine	Tuberomammillary nucleus (TMN)	Sustains wakefulness through widespread cortical activation	Not reported	Not reported
Adenosine	VLPO, basal forebrain	Builds sleep pressure; promotes VLPO GABAergic neuron activity	Not reported	Not reported
Glycine	Medulla, spinal cord, pons	Contributes to REM atonia and supports sleep stability	Not reported	Not reported
Galanin	VLPO, MnPO (preoptic hypothalamus)	Promotes NREM sleep; inhibits arousal	Not reported	Not reported

## LIST OF ABBREVIATIONS

exo-MLT: Exogenous Melatoni
GABA: Gamma-Aminobutyric Acid
NREM: Non-rapid Eye Movement
REM: Sleep and Rapid Eye Movement
SCN: Suprachiasmatic Nucleus

## SUPPLEMENTARY MATERIAL

PRISMA checklist is available as supplementary material on the publisher’s website along with the published article.

## APPENDIX

**Table 1:** Studies on the relationship between exo-MLT and GABAergic system.

**Author(s)**	**Year**	**Tissue or Cell Culture/** **Animal Specie/Patients**	**Study Aim**	**Main Findings/Hypotheses**
***In vitro/ex vivo *Studies**				
Lowenstein *et al*. [46]	1985	Male Wistar rat cerebral cortex membranes	To study exo-MLT affinity for GABA-A receptor expressed in rat cerebral cortex membranes	Exo-MLT at 100 μM reduced (3H)flunitrazepam binding in rat cerebral cortex membranes *in vitro*
Cheng *et al*. [48]	2012	Hippocampal neurons of Wistar rats	To analyse the effects of exo-MLT on GABA-induced current (IGABA) and on GABAergic miniature inhibitory post-synaptic currents (mIPSCs)	MLT (0.1-5 mM) enhanced IGABA and peak amplitude with GABA co-administration, an effect reversed by flumazenil
Yang *et al*. [47]	2021	Dorsal raphe nucleus (DRN) neurons from Sprague-Dawley rats	To explore whether MLT affects DRN serotoninergic neurons *via *mechanisms including inhibitory GABAergic activity.	MLT (10 μM) upregulated NKCC1 in DRN neurons, increasing intracellular Cl- currents and reducing inhibitory GABAergic activity
***In vivo *Studies**				
Lowenstein *et al*. [46]	1985	Adult male Wistar rats	To investigate whether pineal gland removal or MLT treatment affects GABA-A receptor benzodiazepine sites in the cerebral cortex	Pinealectomy reduced GABA-A receptor benzodiazepine sites, reversed by exo-MLT (800-1600 μg/kg). Daily MLT administration for 5 days increased these sites in normal rats
Acuña Castroviejo *et al*. [49]	1986	Adult male Wistar rats	To assess changes in GABA-A receptor binding sites after pinealectomy and MLT administration	Pinealectomy increased GABA-A receptor binding sites, reversed by MLT at a minimal dose of 25 μg/kg
Rosenstein and Cardinali [21]	1986	Adult male Wistar rats	To assess the effect of MLT on the *in vivo* GABA turnover in different rat brain regions	Exo-MLT (25-300 μg/kg) increased GABA accumulation in the rat hypothalamus, cerebellum, cerebral cortex, and pineal gland
Levesque and Locke [55]	1994	Male Fischer rats	To evaluate the discriminative stimulus effects of MLT and other sedative substances	MLT-induced discriminative stimulus (150 mg/kg I.P.) involved GABA-A receptor sites distinct from benzodiazepine sites
Atsmon *et al*. [68]	1996	Male CD strain rats	To assess MLT and diazepam (GABA-A receptor agonist) binding in the medulla-pons and cortex	Chronic diazepam administration inhibited MLT binding sites in the medulla-pons, but this inhibition was reversed with exo-MLT administration (4 mg/L)
Langebartels *et al*. [54]	2001	Male Wistar rats	To explore the effects of MLT and the role of GABA-A receptors on sleep	MLT (5 and 10 mg/kg) evoked a moderate decrease of low EEG frequency activity in non-REM sleep, and this effect was blocked by the GABA-A receptor antagonist picrotoxin
Kolker *et al*. [57]	2002	Male golden hamsters	To determine if incrementing exo-MLT administration could increase the circadian response to triazolam	Chronic MLT administration (9 μg/mL) increased triazolam phase shifting effects in both young and old hamsters
Wang *et al*. [56]	2003	Male Wistar rats	To examine if GABA-A receptors mediate the hypnotic activity of MLT	MLT at 10mg/kg dosage showed sleep improvements in rats. These effects were blocked by the GABA-A receptor antagonist picrotoxin
Kumar *et al*. [53]	2009	Albino Laca strain mice	To study the possible GABAergic mechanisms involved in MLT protective effects in sleep-deprived mice	MLT pre-treatment (5 and 10 mg/kg) was neuroprotective against sleep deprivation-induced behavioural alterations and oxidative stress, and Flumazenil blocked MLT effect
Matsuta *et al*. [59]	2009	Female Sprague-Dawley rats	To test if MLT can decrease nocturia *via *the CNS GABAergic system	MLT (0.43 to 43 pmol) increased in a dose dependent manner bladder capacity, through GABA-A receptor
Hong *et al*. [50]	2016	Male CD-1 mice	To study the relationship between MLT and dopamine receptor signalling sleep induced by pentobarbital, and the cortical intracellular mechanisms of sleep maintenance	MLT decreased sleep latency and increased pentobarbital-induced sleep duration in a dose-dependent manner, with best results at 30 mg/kg dosages, *via *the GABAergic system. MLT did not seem to modify locomotor activity
Zhang *et al*. [61]	2017	Adult Sprague-Dawley rats	To study the effects of MLT in an anxiety model induced by sleep deprivation	MLT at 15mg/kg led to a decrease in corticosterone levels in rats and it reversed GABA-A- α-2 receptor down-regulation in the amygdala, while it had no effects on the expression of GAD67
Huang *et al*. [51]	2020	Adult GAD-67-GFP transgenic and male C57/ BL6 mice	To assess the role of MLT in GABAergic neurons activity in the lateral hypothalamus	In the lateral hypothalamus, MLT (1 μM) suppressed GABAergic neurons activity *via *MT1 activation, and it depended on hyperpolarization-activated cyclic nucleotide-gated (HCN) ion channels inhibition
Borsani *et al*. [58]	2022	Male BTBR T+Itpr3tf/J and male C57BL6/J mice	To analyse the potential positive effects of exo-MLT in an ASD model	Early chronic administration of exo-MLT (10 mg/kg) ameliorated ASD dysfunction in the brain. It produced a decrease in glutamatergic mGluR7/8 receptors expression and a decrease in glutamate decarboxylase enzyme GAD67
Xu *et al*. [60]	2023	Male inbred C57BL/6JNifdc mice	To investigate the effects of MLT in a mice model of PTSD	MLT at 2 mM concentration alleviated stressful behaviours and restored both GABA and cortisol levels through MT1 receptors
**Human studies**				
Ferini-Strambi *et al*. [62]	1993	Healthy male subjects	To study the effect of MLT on polysomnography and some microstructural parameters, with or without triazolam	100 mg of MLT with or without triazolam did not alter polysomnography, but modulated microstructural parameters, such as arousal during sleep. Subjective sleep quality was increased in both MLT and MLT+triazolam conditions
Cruz-Aguilar *et al*. [32]	2018	Patients with Alzheimer’s Disease (AD)	To examine MLT effect on cortical activity during sleep by electroencephalographic recording	MLT (5mg) decreased sleep latency in patients with AD by modulating some electroencephalographic parameters, such as alpha 1 and delta bands

**Table 2:** Studies on the relationship between exo-MLT and serotoninergic system.

**Author(s)**	**Year**	**Tissue or Cell Culture/** **Animal Specie/Patients**	**Study Aim**	**Main Findings/Hypotheses**
***In vitro/ex vivo *Studies**				
Iyengar *et al*. [72]	1994	Skin samples from people with vitiligo	To explore the effects of the exo-MLT on melanocytes function in relation to light exposure	Melanocytes’ response to UV exposure was mediated by 5-HT and MLT activity. Higher 5-HT levels corresponded to short nights and long days, conversely, higher MLT levels corresponded to long nights and short days
Muneoka *et al*. [70]	2000	Striatum and hippocampus of male and female Sprague-Dawley rats	To explore the effects of the exo-MLT in the neonatal period on serotonin	Neonatal administration of MLT increased 5-HT and 5-hydroxy-3-indolacetic acid (5-HIAA) levels in the striatum and hippocampus of 5-HIAA
Monnet *et al*. [22]	2002	Hippocampal slices from adult male Sprague-Dawley rats	To explore the effect of MLT on the circadian serotoninergic transmission in the hippocampus	MLT decreased spontaneous and evoked (3H)5-HT efflux in a dark phase, while it increased (3H)5-HT in presence of light. Nifedipine could inhibit MLT effects only in light phases
Pazo *et al*. [71]	2002	Pineal gland of adult male Wistar rats	To investigate the effects of exo-MLT on 24-hour rhythms of norepinephrine and 5-HT on young and old animals	MLT (30 μg) incremented the 24-hour rhythm amplitude of norepinephrine and doubled the pineal 5-HT turnover especially in young rats, but not in old ones
Bassani *et al*. [69]	2014	Striatum of male Wistar Rats	To study the effects of chronic exo-MLT administration in a rotenone-induced model of PD	Increase of 5-HT (and dopamine) levels in the striatum of rats treated with MLT, possibly by inhibiting MAO activity
Yang *et al*. [47]	2021	Dorsal raphe nucleus (DRN) neurons from Sprague-Dawley rats	To study the effect of MLT on the DRN serotoninergic neurons	MLT through its receptors increased tryptophan hydroxylase 2 (TPH2) expression in DRN serotoninergic neurons and influenced wakefulness by the modulation of WNK-SPAK/OSR1-KNCC1 signalling, mediated by NO
***In vivo *Studies**				
Dugovic *et al*. [78]	1989	Adult male Wistar rats	The study the interaction between MLT and two serotoninergic compounds on sleep pattern	MLT at 1 mg/kg showed no effects on sleep-wake state. When administered with ritanserin (5-HT 2 antagonist) or DOM (5-HT partial agonist), MLT can counteract their effects, probably by indirectly modulating 5-HT2 receptors
Oxenkrug *et al*. [74]	2003	Various strains of male rats	To understand the role of exo-MLT and N-acetyl5-HT(NAS) administration on jet lag	MLT (1 mg/kg) did not restore physiologic serotonin, MLT, and NAS rhythms in SHR rats, it did not restore serotoninergic pineal rhythm, but was able to restore MLT and NAS rhythms in Sprague-Dawley rats, and was able to restore serotoninergic, melatoninergic, and NAS rhythms in WKY rats
Paredes *et al*. [75]	2007	Male and female ringdoves (Streptopelia risoria)	To explore the effect of various MLT dosages in young and older diurnal animals on 5-HTand MLT levels	Exo-MLT (2.5 and 5 mg/kg) decreased older and younger animals’ activity. For older ringdoves the effect was evident especially during the night, while for the young ones it was present in nocturnal and diurnal activity, depending on the dose. 5-HT levels were unaffected by exo-MLT
Paredes *et al*. [76]	2009	Male and female ringdoves (Streptopelia risoria)	To study the effect of MLT or tryptophan administration on circadian rhythms in older animals	MLT (0.25-2.5 mg/kg) was able to increase MLT levels in old animals, lowered nocturnal activity, core temperature, and cytokine levels. 5-HT levels were not affected
Jagota & Kalyani [79]	2010	Male Wistar rats	To study the effect of aging of exo-MLT on 5-HT rhythms in rats of different age	The administration of exo-MLT (30 μg/kg) restored the serotoninergic levels in younger rats, while there was a loss of MLT sensitivity in older rats
Chenu *et al*. [73]	2013	Male Sprague-Dawley rats	To investigate the effects of MLT and 5-HT antagonists in various brain regions	Daily MLT administration at 40 mg/kg alone or in combination with 5-HT antagonists for two weeks showed no effect on DRN serotoninergic firing and on VTA dopaminergic firing
Haduch *et al*. [77]	2016	Male Wistar-Han rats	To assess if MLT administration mediates 5-HT synthesis	MLT injection (100 mg/kg) increased CYP2D-mediated synthesis of 5-HT from MLT-derived 5-MT *in vivo* *via *deacetylation
Wang *et al*. [81]	2019	Male IRC mice	To study the effects of MLT on aggressive behaviours on methamphetamine-induced aggressiveness model	MLT treatment (5 mg/kg) reversed methamphetamine-induced aggressive behaviours, prolonging onset latency. MLT increased the concentration of 5-HIAA, dopamine, and homovanillic acid after methamphetamine treatment
Poceviciute *et al*. [80]	2022	Male and female Wistar rats	To examine the effects of MLT administration in an animal model of AUD	The administration of MLT (40 mg/kg) reduced alcohol intake in rats treated also with escitalopram
Xu *et al*. [60]	2023	Male inbred C57BL/6JNifdc mice	To investigate the effects of MLT in a mice model of PTSD	MLT at 2 mM concentration alleviated stressful behaviours and restored both GABA and cortisol levels, through MT1; conversely, 5-HT levels were not altered

**Table 3:** Studies on the relationship between exo-MLT and dopaminergic system.

**Author(s)**	**Year**	**Tissue or Cell Culture/** **Animal Specie/Patients**	**Study Aim**	**Main Findings/Hypotheses**
***In vitro/ex vivo *Studies**				
Schiller *et al*. [82]	2003	Pheochromocytoma cell line (PC12)	To study the relationship between MLT and dopamine release induced by nicotine	MLT inhibited dopamine release elicited by nicotine in PC12 cells in an acute setting, but not during chronic exposure
***In vivo *Studies**				
Holmes and Sugden [83]	1982	Male Sprague-Dawley rats	To investigate the effects of exo-MLT on the sleep-wake cycle, and changes in catecholamines concentration	MLT at 10 mg/kg (and partially at 2.5 mg/kg) reduces sleep onset time and wakefulness. At 20 mg/kg, it does not affect brain dopamine or other catecholamine levels
Matsumoto and Ducsay [84]	1992	Rhesus macaque	To investigate if MLT administration restore a normal catecholamine pattern and day-night cycle in pregnant macaque under constant light exposure	MLT infusion during the night (0.2 pg/kg/h) in a setting of constant light did not seem to modify epinephrine and dopamine rhythms
Zhdanova *et al*. [85]	2002	Male Sprague-Dawley rats	To test the effects of MLT administration in rats by measuring cAMP level changes after acute cocaine injection and MLT pre-treatment.	MLT pre-treatment can significantly reduce cAMP increase induced by cocaine in the NAcc of rats, although it is not able to completely restore the pre-cocaine cAMP levels
Bassani *et al*. [69]	2014	Male Wistar rats	To study the effects of exo-MLT in a rotenone-induced model of PD	MLT treatment prevented depressive-like states in rotenone-treated rats, increasing dopamine levels in the striatum by MAO inhibition
Chenu *et al*. [73]	2014	Male Sprague-Dawley rats	To investigate the effects of repeated administrations of MLT and of serotonin antagonists, alone and in combination, in various brain regions	Daily MLT administration at 40 mg/kg alone or in combination with serotonin antagonists enhanced the number of spontaneous firings in dopaminergic neurons in the ventral tegmental area
Haduch *et al*. [77]	2016	Male Wistar-Han rats	To assess if MLT administration supports CYP2D-mediated serotonin and dopamine synthesis	MLT injection (100 mg/kg) did not change the extracellular dopamine concentration in naïve rats in the striatum, but if rats are pre-treated with pargyline there is an increase of up to 500% of dopamine
Hong *et al*. [50]	2016	Male CD-1 mice	To study the relationship between MLT and dopaminergic D2-receptor signalling sleep induced by pentobarbital	MLT decreases sleep latency and increases pentobarbital-induced sleep duration in a dose-dependent manner, with best results at 30 mg/kg dosages. Quinpirole is able to increase the length of MLT-augmented sleep
Wang *et al*. [88]	2019	Male IRC mice	To study the effects of MLT on aggressive behaviours and on neurotransmitters in a methamphetamine-induced aggressiveness model	MLT treatment (5 mg/kg) reversed methamphetamine-induced aggressive behaviours and normalize homovanillic acid/dopamine, (3,4-dihydroxyphenyl acetic acid + homovanillic acid)/dopamine and 5-HIAA/serotonin ratios
Hussain *et al*. [88]	2020	Sprague Dawley rats	To study the effects of haloperidol (D2 antagonist) on hypokinesia in light and dark phases, and to investigate the effects of MLT	MLT (15 mg/kg) is able to reduce haloperidol-induced hypokinesia in adult rats, but not in adolescent animals. Older rats had a natural decrease in MLT, and were not affected by modifications in lightning
Sumaya and Dubocovich [87]	2022	C57BL/6 mice	To investigate the effects of exo-MLT in a hypokinesia model induced by fluphenazine in light and dark phases	MLT (10 mg/kg) can reduce hypokinesia in knockout and wild type mice, while it does not seem to counteract hypokinesia during the dark phase
Aranda-Martínez *et al*. [86]	2023	Zebrafish (*Danio rerio*)	To study MLT effects on the MPTP model of PD	Administration of exo-MLT reversed the dopaminergic impairment and restored day/night cycles
Xu *et al*. [60]	2023	Male inbred C57BL/6JNifdc mice	To investigate the effects of MLT in mice behaviour in a PTSD model	MLT (2 mM) was not able to modify significantly dopamine levels

**Table 4:** Studies on the relationship between exo-MLT and glutamatergic system.

**Author(s)**	**Year**	**Tissue or Cell Culture/** **Animal Specie/Patients**	**Study Aim**	**Main Findings/Hypotheses**
***In vitro/ex vivo *Studies**				
Evely *et al*. [91]	2016	Lateral habenula neurons from male Sprague-Dawley rats	To evaluate the effects of exo-MLT on Glu transmission and neuronal excitability in medial lateral habenula	Exo-MLT increased Glu synaptic firing and the neuronal excitability of the lateral habenula neurons
***In vivo *Studies**				
Appelbaum *et al*. [97]	2009	Zebrafish (Danio rerio)	To examine the role of MLT in regulating the sleep-wake cycle in fishs lacking HCRT receptors (HCRTR-/-)	In HCRTR^−/−^ zebrafish, exo-MLT administration increased sleep duration by 30% and sleep consolidation (sleep-wake transitions) by 40%
Tardito *et al*. [92]	2010	Sprague-Dawley rats	To evaluate the effects of MLT, agomelatine, and a 5-HT antagonist on stress-induced Glu release	Exo-MLT was not able to decrease stress-induced Glu increments
Zhang *et al*. [61]	2017	Adult Sprague-Dawley rats	To study the effects of MLT in an anxiety model induced by sleep deprivation	MLT at 15mg/kg decreased NR2B-containing N-methyl-D-aspartate and GluR1 receptors up-regulation
Mao *et al*. [93]	2021	SPF male C57BL/6 mice	To examine the role of MLT in chronic inflammation	Exo-MLT was able to reduce the necroptosis-related Glu metabolic pathway
Borsani *et al*. [58]	2022	Male BTBR T+Itpr3tf/J and male C57BL6/J mice	To analyse the potential effects of exo-MLT in an ASD model	Early chronic administration of exo-MLT (10 mg/kg) ameliorated ASD dysfunction in the brain. It produced a decrease in glutamatergic mGluR7/8 receptors expression and Glu decarboxylase enzyme GAD67
Zhao *et al*. [94]	2024	Male Sprague-Dawley rats	To examine the role of exo-MLT and exercise in a rat model of ischemic stroke	Exo-MLT in combination with exercise improved GluR2 expression and reduced Glu excitotoxicity

**Table 5:** Studies on the relationship between exo-MLT and norepinephrine/epinephrine systems.

**Author(s)**	**Year**	**Tissue or Cell Culture/** **Animal Specie/Patients**	**Study Aim**	**Main Findings/Hypotheses**
***In vivo *Studies**				
Chuluyan *et al*. [95]	1991	Wistar rats	To study the effects of MLT on the release and uptake of norepinephrine (NE)	MLT (300 µg/kg) administered at the onset of darkness reduced NE turnover by 40%. *In vitro*, it inhibited depolarization-induced NE release only at night (10^-8^-10^-6^ M) and reduced uptake at low concentrations (0.5 pM)
Matsumoto and Ducsay [84]	1992	Rhesus macaques (Macaca mulatta)	To investigate the effects of photoperiod and MLT infusion on plasma catecholamine levels during late gestation.	Exo-MLT infusions (0.2 µg/kg/h) under constant light did not restore the rhythm of NE or epinephrine (EPI) secretion
Brusco *et al*. [96]	1998	Sprague-Dawley rats	To examine exo-MLT on the 24-hour rhythms of NE and acetylcholine synthesis	MLT treatment (10 or 100 μg for 17 days) enhanced NE synthesis
Pazo *et al*. [71]	2002	Wistar rats	To evaluate the effect of MLT treatment on 24-hour rhythms of NE content and serotonin turnover in the pineal gland	MLT treatment (30 µg/die for 11 days) increased the amplitude of the 24-hour rhythm of pineal NE content by 120% in young rats and by 52% in old rats
Chenu *et al*. [73]	2013	Sprague-Dawley rats	To study the effects of MLT administration on the activity of dopaminergic (DA), NE and serotonin (5-HT) neurons	MLT administration (40 mg/kg/day for 14 days) had no significant effects on the frequency and pattern of firing of NE neurons in the locus coeruleus
Xu *et al*. [60]	2023	C57BL/6JNifdc mice	To study the effects of MLT on PTSD-like behaviors and levels of cortisol, GABA, and EPI	Exo-MLT (2 mM) reduced EPI and NE levels and alleviated PTSD-like symptoms

**Table 6:** Studies on on the relationship between exo-MLT and orexinergic system.

**Author(s)**	**Year**	**Tissue or Cell Culture/** **AnimalSpecie/Patients**	**Study Aim**	**Main Findings/Hypotheses**
***In vivo *Studies**				
Appelbaum *et al*. [97]	2009	Zebrafish (Danio rerio)	To examine the role of orexin (HCRT) and MLT in regulating the sleep-wake cycle in fishs lacking HCRT receptors (HCRTR^-/-^)	In HCRTR^−/−^ zebrafish, exo-MLT administration increased sleep duration by 30% and sleep consolidation (sleep-wake transitions) by 40%
Appelbaum *et al*. [98]	2010	Zebrafish (Danio rerio)	To study the interaction between the orexin system and MLT, in orexin neurons overexpressing the NPTX2b gene.	Overexpression of NPTX2b in HCRT neurons increased axonal synaptic density and induced resistance to exo-MLT
Sharma *et al*. [23]	2018	Adult male C57BL/6J mice	To evaluate whether MLT promotes sleep by inhibiting orexin neurons	MLT infusion increased NREM sleep by 43.7% and reduced wakefulness by 12.3%, with a significant 66% reduction in active orexinergic neurons. The MLT receptor antagonist luzindole increased wakefulness and reduced NREM and REM sleep
Mi *et al*. [25]	2020	Zebrafish (Danio rerio)	To examine how exposure to 17β-trenbolone influences sleep-wake behavior and evaluate the protective potential of MLT	MLT counteracted the pro-wakefulness effects induced by 17β-trenbolone. MLT administration reduced the expression of HCRT and HCRTR-2, returning them to normal levels and attenuating the insomnia-like phenotype
Cakir *et al*. [99]	2023	Male Sprague Dawley rats	To investigate how REM sleep deprivation in rats influences leptin, nesfatin-1 and orexin-A levels in rats, and to evaluate the effect of MLT treatment	MLT influenced leptin and nesfatin-1 levels, but it had no significant effect on orexin-A levels

**Table 7:** Studies on on the relationship between exo-MLT and ACh system.

**Author(s)**	**Year**	**Tissue or Cell Culture/** **AnimalSpecie/patients**	**Study Aim**	**Main Findings/Hypotheses**
***In vivo* Studies**				
Brusco *et al*. [96]	1998	Sprague-Dawley rats	To examine exo-MLT on the 24-hour rhythms of norepinephrine and acetylcholine synthesis	MLT treatment (10 or 100 μg for 17 days) enhanced ACh and NorEpi synthesis
Cardinali *et al*. [101]	1998	Sprague-Dawley rats	To study MLT activity during circadian rhythms in lymphnodes and spleen in an immune-mediated inflammation model in rats	Exo-MLT increased daily oscillations of 3(H)ACh production in young and especially in old rats
Paredes *et al*. [137]	1999	Adult male Sprague–Dawley rats	To examine the effects of exo-MLT on extracellular ACh in the NAcc and motor activity	Exo-MLT dose-dependently increased ACh concentrations in the NAcc
de Prado *et al*. [102]	2003	Male Wistar rats	To evaluate whether MLT perfused in the medial PFC could vary ACh levels and locomotor activity	Exo-MLT disrupted ACh circadian rhythm in a dose dependent manner
Tancheva *et al*. [103]	2021	Male Wistar rats	To study the effects of exo-MLT pretreatment in a 6-OHDA PD model and in healthy rats	Exo-MLT increased ACh levels in a PD model

**Table 8:** Studies on the relationship between exo-MLT and adenosinergic system.

**Author(s)**	**Year**	**Tissue or Cell Culture/** **Animal Specie/Patients**	**Study Aim**	**Main Findings/Hypotheses**
***In vitro/ex vivo* Studies**				
Cao *et al*. [106]	2021	Retina of common goldfish (*Carassius auratus*)	To study the retinal circadian clock and the possible interconnections between adenosine, MLT and dopamine in its regulation	Dark-adapted neural retinas, perfused with MLT (100 nM) during the subjective day or with the MLT receptor antagonist luzindole (10 µM) during the subjective night, do not show any effects on adenosine content or overflow
von Gall *et al*. [104]	2002	Hypophyseal pars tuberalis (PT) of adult male C3H/HeN mice and male Siberian hamsters	To explore the way that peripheral cells can interpret rhythmic signals that are disseminated from the central circadian clock	MLT (1 µg/g) administrated in Siberian hamsters pre-pinealectomy preserves the expression or *Per1* mRNA and PER1 protein in the PT. The effect of exo-MLT is mediated by adenosine A2b receptor signalling and clock-gene expression
Zamorskii and Pishak [105]	2003	Forebrain of juvenile male mongrel albino rats	To examine the effects of exo-MLT administration on 5’-nucleotidase activity, which is the synthetising enzyme of adenosine in tissue exposed to acute hypobaric hypoxia	MLT (1 mg/kg) does not change 5’-nucleotidase levels in animal tissues from rats exposed to normal lightning conditions. In an acute hypoxia setting, MLT can increase 5’-nucleotidase levels by 18%. In tissue from normoxic (but not hypoxic) animals kept in constant darkness, exo-MLT promotes adenosine synthesis by 23%
